# Influence of vegetation area and edge length on mammals in urban woods

**DOI:** 10.1080/19768354.2017.1348983

**Published:** 2017-07-13

**Authors:** Eun-Jae Lee, Shin-Jae Rhim

**Affiliations:** a Urban Planning Research Group, Daejeon Sejong Research Institute, Daejeon, Korea; b School of Bioresource and Bioscience, Chung-Ang University, Ansung, Korea

**Keywords:** Conservation, field sign, fragmented urban landscape, frequency, species richness

## Abstract

We investigated the relationships between vegetation area, edge length, and mammals in the urban woods of Daejeon Metropolitan City, South Korea. The vegetation patches included in this study varied from 2.1 to 501.0 ha in size. Surveys were conducted monthly between February and October 2015, with a survey route measuring 1 km in length and 10 m width established in each forest patch. Field signs of 14 species of mammals were recorded in the 33 chosen forest patches over the course of the study period, and the number of species in each patch varied from 2 to 11. Mammal species richness was positively correlated with vegetation area, and field sign frequency was positively correlated with vegetation area and negatively correlated with edge length. The field sign frequencies of large moles *Mogera robusta*, Siberian chipmunks *Tamias sibiricus*, leopard cats *Prionailurus bengalensis*, Korean hares *Lepus coreanus*, water deer *Hydropotes inermis*, and wild boars *Sus scrofa* were positively correlated with vegetation area. Moreover, that of large moles, leopard cats, Korean hares, and water deer were negatively correlated with edge length. Remnant vegetation area and edge length are the primary determinants of mammal species richness and field sign frequency in urban woods, highlighting the importance of vegetation patch size for mammal conservation in fragmented urban landscapes.

## 1. Introduction

Human activities have led to dramatic changes and disturbances to natural habitats in many regions of the world (Rosenblatt et al. 1999), with habitat loss and fragmentation now recognized to be a major threat to global biodiversity (Laurance and Bierregaard 1997; Pardini et al. 2005). Habitat fragmentation decreases habitat size while increasing both patch edge length and distances between patches (Lee et al. 2017). Habitat fragmentation has different influences on different species. In general, interior species richness decreases and edge species richness increases overall with fragmentation (Laidlaw 2000).

Loss and fragmentation of habitats is the main driver of the biodiversity crisis in urban landscapes. Moreover, the original forests have been converted into new anthropogenic vegetated patches (Foley et al. 2005; Estavillo et al. 2013). Species richness and abundance typically decline as a result of habitat fragmentation, at least until populations adjust to the size of the habitat (de Castro and Fernandez 2004; Lee et al. 2014). Moreover, micro- and macro-climatic changes increase in plant mortality, regeneration of vegetation, increase of predation risk, and invasion by other species often occur in response to habitat fragmentation (Umapathy and Kumar 2000; Barrantes et al. 2016). In urban woods, these changes would be expected to influence mammal species and their habitats.

Identifying factors that increase the vulnerability of mammal populations is important for minimizing the loss of mammals and their habitats in fragmented areas (Laurance 1991; Laurance and Bierregaard 1997). Expanding urbanization has altered species composition, abundances of individuals, size and shape of habitats, and landscape pattern in urban landscapes (Lee et al. 2017). Although fragment size serve as effective predictors of species occurrence in forest patches (Park and Lee 2000; Umapathy and Kumar 2000), more detailed studies are needed for conservation of biodiversity in urban woods.

With its urban population reaching 90% of the total human population, South Korea is one of the most urbanized countries in the world (Korea Land & Housing Corporation 2017). Studies on urban ecology are needed for the conservation of species and their habitats in urbanized areas. However, there are very few studies about the influence of urbanization on mammals within urban woods in South Korea. In this study, we explored how mammal species respond to urban woods fragments.

Surveys of mammalian field signs, being comparatively easy to conduct, thus are an effective and efficient means of examining the characteristics of mammal communities displaced by increasing habitat fragmentation in urban landscapes. The objective of this study was to investigate the relationships between vegetation area, edge length, and mammals in the urban woods of Daejeon, South Korea. We analyzed (1) the patterns of relative field sign frequency of mammals and (2) relationships between vegetation area or edge length of urban woods and richness and frequency of mammals.

## 2. Methods

The study was carried out from February to October 2015 in the urban woods of Daejeon Metropolitan City (36°17′–27′N, 127°17′–28′E), South Korea. Daejeon is one of the largest cities in South Korea. In Daejeon, urbanization has progressed rapidly since the 1970s. The transformation and deforestation of natural habitats into urbanized areas has resulted in a mosaic of patch types, including heavily built downtown and semi-natural habitats. Most of the remaining vegetation is composed of small isolated patches. The annual mean temperature is 14.2°C (range − 10.6–36.8°C) and annual precipitation is 883 mm. The metropolitan area of Daejeon encompasses 540, of which 286 km^2^ is vegetated. There are 603 vegetated patches in the urban woods of Daejeon, ranging from 1 to 1934 ha (Daejeon Development Institute 2015).

Thirty-three vegetated patches were selected for inclusion in the survey (Table 1). The patches were selected by area: 6 patches of 1–10ha, 8 patches of 11–20 ha, 3 patches of 21–30 ha, 4 patches of 31–40 ha, 1 patch of 50–100 ha, 3 patches of 100–200 ha, 7 patches of 200–500 ha, and 1 patch of 500–1000 ha. Large patches over 1000 ha (Sajeong Park: 1482 ha and Daejeon National Cemetery: 1934 ha) were excluded as study sites because their vegetation area was too large for proper comparison. The 33 studied patches ranged in area from 2.1 to 501.0 ha. The area and edge length of each patch was measured with a land cover map using ArcGIS 10.0 (ESRI Inc., Redlands, CA, USA). Survey routes measuring 1 km in length and 10 m width were established in each of the 33 patches.

**Table 1.** Vegetation area, edge length and number of mammal species observed via field signs in the urban woods of Daejeon, South Korea.

Site no.	Site name	Area (ha)	Edge length (km)	No. of mammal species
1	Ojeong Farm Market	2.1	3.4	2
2	Byeondong Park	2.6	0.7	4
3	Panamdong South	4.1	1.0	3
4	Hannam University	8.8	2.5	4
5	Daejeong Elementary School	9.1	2.1	3
6	Daejeon/Woosong University	9.5	2.2	6
7	Keonyang University Hospital	11.0	2.1	4
8	Daeshin High School	11.1	1.6	4
9	Namsun Park	12.2	1.8	3
10	Yongjeon Park	15.3	3.4	4
11	Kwanpyeongdong	16.7	3.5	6
12	Daejeon University	18.1	2.5	6
13	Yeojin Buddhism Museum	18.9	2.8	3
14	Cheongbyeoksan Park	19.9	1.9	6
15	Saesomang Church	24.5	3.6	4
16	Humansia Apartment	25.6	4.1	5
17	Seongdusan Park	26.2	2.7	6
18	Hoedeok Park	31.5	2.3	6
19	Panamdong North	34.0	4.1	6
20	Maebong Park	41.5	3.0	7
21	Woosong University	46.6	4.5	6
22	Eulmigi Park	58.2	7.3	6
23	Chungnam National University	101.4	8.5	11
24	Jangan Reservoir	137.0	4.7	11
25	Doan Park	151.0	13.8	5
26	Yongho Public Cemetery	203.0	6.1	9
27	Songlim Temple	218.0	7.7	10
28	Bokyong Horse Racing Course	220.0	8.0	10
29	Hyemyeong Temple	223.0	9.1	7
30	Gasuwon Park	308.7	13.5	10
31	Obongsan	344.0	16.9	9
32	National Research Institute of Cultural Heritage	413.0	21.5	9
33	Wolpyeong Park	501.0	15.2	8

We counted the field signs of the following mammal species along the survey routes in the established vegetation transects in the urban woods: large mole *Mogera robusta*, Asiatic chipmunk *Tamias sibiricus*, Eurasian red squirrel *Sciurus vulgaris*, Siberian flying squirrel *Pteromys volans*, raccoon dog *Nyctereutes procyonoides*, domestic dog *Canis lupus familiaris*, leopard cat *Prionailurus bengalensis*, domestic cat *Felis catus*, Eurasian badger *Meles meles*, Siberian weasel *Mustela sibirica*, yellow-throated marten *Martes flavigula*, Korean hare *Lepus coreanus*, water deer *Hydropotes inermis*, and wild boar *Sus scrofa*. Field signs included tracks, roosts, pellets, droppings, and skulls in this study.

We performed 9 days (1 day/month × 9 months) of tracking on each of 33 survey routes from February to October in 2015. We also noted incidental sightings of mammal species on the survey routes during the survey. The number of field signs recorded per sampling day on each survey route was established as field sign frequency of mammals. We did not count the field signs that were difficult to distinguish by species. We determined the mammal species richness based on the total number of species detected using all of the field signs (Rhim and Lee 2007; Hwang et al. 2014; Son et al. 2017).

Statistical analyses were performed using the SPSS statistical package for Windows. Multiple regression (Poisson regression) analysis based on a generalized linear model (GLM) was used to examine the relationships among vegetation area, edge length and the variables of interest (specifically mammal species richness and field sign frequency). The vegetation area (ha) and edge length (km) were log-transformed to perform multiple regressions. Multiple regression analysis was also employed to examine the relationships between vegetation area, edge length, and the field sign frequency of each mammal species. Akaike Information Criterion (AIC) model weights (*ω*) were determined for each of the variables that were present in at least one selected model resulting from the generalized linear model with Poisson distribution. Values were considered statistically significant at *p* < .05.

## 3. Results

Field signs of 14 mammal species were recorded over the course of the study period, with the number of species per patch varying from 2 to 11 in the 33 vegetation patches (Table 2). A total of 1261 mammalian field signs were observed in this study. Although field sign frequency varied greatly within and among patches, water deer, raccoon dogs, Asiatic chipmunks, and large moles were determined to be the predominant mammals. The field signs of several endangered species, such as Siberian flying squirrels, leopard cats, and yellow-throated martens, were observed (Table 2).

**Table 2.** Mean numbers of monthly observed mammalian field signs (ea/ha) in the urban woods, Daejeon, South Korea. Field signs were surveyed at each site once every month from February to October 2015. Site numbers as in Table 1.

	Species^a^														
Site no.	MR	TS	SV	PV	NP	CL	PB	FC	ML	MM	MS	LC	HI	SS	Total frequency
1					0.11	0.33									0.44
2					0.11	0.33				0.11			0.56		1.11
3					0.33	0.22		0.11							0.66
4		0.11			0.11	0.33							0.56		1.11
5						0.33		0.22		0.11					0.66
6	0.11	0.11			0.22	0.22				0.11			0.89		1.66
7					0.22	0.33		0.22					0.67		1.44
8					0.11	0.44		0.11					1.11		1.77
9					0.22	0.33							1.00		1.55
10		0.44			0.22	0.11							1.67		2.44
11	0.22				1.00	0.56		0.11		0.11			1.78		3.78
12	0.11	0.22			0.22	0.11				0.11			1.56		2.33
13					1.00	0.56							1.33		2.89
14	0.33		0.11		0.89	0.33				0.11			1.56		3.33
15		0.22			0.89	0.44							1.78		3.33
16		0.11			0.33	0.22		0.11					1.00		1.77
17		0.33			0.78	0.44		0.22		0.22			1.67		3.66
18	0.22	0.22			0.67	0.22				0.11			1.44		2.88
19	0.22	0.22			0.67	0.22				0.22			1.00		2.55
20	0.44	0.33			0.78	0.11		0.11		0.11			1.89		3.77
21	0.44	0.22	0.11		0.56			0.11					1.44		2.88
22	0.33	0.56			1.22	0.33						0.11	1.44		3.99
23	1.22	0.89			1.44	0.11	0.11	0.11		0.33	0.11	0.22	2.56	0.33	7.43
24	0.67	1.11	0.22		0.56		0.44		0.11	0.22	0.22	0.22	2.44	0.44	6.65
25		0.22			0.22	0.33		0.22					1.56		2.55
26	0.56	0.78			0.67	0.22	0.33			0.22		0.56	3.44	0.67	7.45
27	0.67	0.89		1.33	0.33		0.22			0.11	0.22	0.22	3.67	0.22	7.88
28	0.78	1.00	0.22		0.44	0.11	0.22			0.22		0.22	2.56	0.22	5.99
29	1.11				0.89		0.33			0.56		0.78	3.00	0.67	7.34
30	1.33	1.22			1.44	0.22	0.22		0.11	0.22		0.33	2.89	0.44	8.42
31	0.78	0.89			0.89		0.33		0.22	0.22		0.22	3.78	0.33	7.66
32	0.44	0.67			0.44	0.56		0.33		0.33		0.11	2.78	0.89	6.55
33	0.67	0.56	0.22		0.44	0.44		0.11		0.22			2.22		4.88

^a^Species – MR: *Mogera robusta*, TS: *Tamias sibiricus*, SV: *Sciurus vulgaris*, PV: *Pteromys volans*, NP: *Nyctereutes procyonoides*, CL: *Canis lupus*, PB: *Prionailurus bengalensis*, FC: *Felis catus*, MM: *Meles meles*, MS: *Mustela sibirica*, MF: *Martes flavigula*, LC: *Lepus coreanus*, HI: *Hydropotes inermis*, SS: *Sus scrofa*.

The best model of mammal species richness in urban woods had an Akaike weight (*ω*) of 0.40–0.59. The top-ranked model for species richness was 0.92 + 0.53 log (vegetation area) with a coefficient of determination (*R*^2^) of 0.53. The second-ranked model contained the log (vegetation area) and log (edge length). The top-ranked model for field sign frequency was 1.72 + 1.32 log (vegetation area) – 0.77 log (edge length) with *R*^2^ = 1.00. It had an Akaike weight (*ω*) of 0.00–0.99. The log (vegetation area) was dominated as a predictor variable in the second-ranked model (Table 3).

**Table 3.** Top-ranked models based on the correlated Akaike Information Criterion (AIC*_c_*) explaining the species richness and field sign frequency of mammals in urban woods, Daejeon, South Korea based on the model selection resulting from generalized linear model with Poisson distribution.

						Intercept		Vegetation area		Edge length	
Variables	Top-ranked model	AIC*_c_*	ΔAIC*_c_*	*ω_i_*	*R*^2^	SE	*p*	SE	*p*	SE	*p*
Species richness	Intercept + log (vegetation area)	133.54	0.00	0.59	0.53	0.210	< 0.001	0.110	< 0.001	–	–
	Intercept + log (vegetation area) + log (edge length)	134.34	0.80	0.40	0.51	0.241	0.001	0.285	0.003	0.504	0.207
Field sign frequency	Intercept + log (vegetation area) + log (edge length)	267.49	0.00	0.99	1.00	0.117	< 0.001	0.129	< 0.001	0.222	< 0.001
	Intercept + log (vegetation area)	277.32	9.83	0.01	1.00	0.101	< 0.001	0.049	< 0.001	–	–
	Intercept + log (edge length)	380.19	112.71	0.00	0.99	0.073	< 0.001	–	–	0.085	< 0.001

Multiple regression (Poisson regression) analysis based on a GLM was used to examine the relationships between vegetation area or edge length and field sign frequency of all 14 mammal species. We determined that the field sign frequencies of large moles, Asiatic chipmunks, leopard cats, Korean hares, water deer, and wild boars were positively correlated with vegetation area. Moreover, the field sign frequencies of large moles, leopard cats, Korean hares, and water deer were negatively correlated with edge length.

The best model of field sign frequency of large moles had an Akaike weight (*ω*) of 0.00–0.61. The top-ranked model field sign frequency of large moles was −2.41 + 2.41 log (vegetation area) – 1.42 log (edge length) with a coefficient of determination (*R*^2^) of 0.93. That of leopard cats was −8.11 + 6.09 log (vegetation area) – 6.01 log (edge length) with *R*^2^ = 0.70 (*ω* = 0.98). Korean hares had − 6.49 + 4.66 log (vegetation area) – 3.86 log (edge length) as the top-ranked model with *R*^2^ = 0.75 (*ω* = 0.87). Moreover, the top-ranked model of field sign frequency of water deer was 1.05 + 1.29 log (vegetation area) – 0.86 log (edge length) with *R*^2^ = 0.98 (*ω* = 0.90). The top-ranked model of Asiatic chipmunks (*R*^2^ = 0.89, *ω* = 0.62) and wild boars (*R*^2^ = 0.88, *ω* = 0.62) contained the log (vegetation area). The log (vegetation area) was dominated as a predictor variable in the second-ranked models of large moles, leopard cats, Korean hares, and water deer with *R*^2^ of 0.59–0.98. Moreover, the second-ranked model of Asiatic chipmunk and wild boar contained the log (vegetation area) and log (edge length) with *R*^2^ of 0.87–0.89 (Table 4).

**Table 4.** Top-ranked models based on the correlated Akaike Information Criterion (AIC*_c_*) explaining the field sign frequency of mammal species in urban woods, Daejeon, South Korea based on the model selection resulting from generalized linear model with Poisson distribution.

						Intercept		Vegetation area		Edge length	
Species	Top-ranked model	AIC*c*	ΔAIC*_c_*	*ω_i_*	*R*^2^	SE	*p*	SE	*p*	SE	*p*
*Mogera robusta*	Intercept + log (vegetation area) + log (edge length)	124.61	0.00	0.61	0.93	0.517	< 0.001	0.482	< 0.001	0.781	0.069
	Intercept + log (vegetation area)	125.52	0.91	0.39	0.92	0.442	< 0.001	0.199	< 0.001	–	–
	Intercept + log (edge length)	149.51	24.90	0.00	0.84	0.289	0.027	–	–	0.302	< 0.001
*Tamias sibiricus*	Intercept + log (vegetation area)	130.38	0.00	0.62	0.89	0.399	< 0.001	0.183	< 0.001	–	–
	Intercept + log (vegetation area) + log (edge length)	131.37	0.99	0.38	0.89	0.459	< 0.001	0.452	< 0.001	0.748	0.232
	Intercept + log (edge length)	148.50	18.11	0.00	0.81	0.271	0.119	–	–	0.289	< 0.001
*Prionailurus bengalensis*	Intercept + log (vegetation area) + log (edge length)	47.68	0.00	0.98	0.70	2.024	< 0.001	1.490	< 0.001	2.019	0.003
	Intercept + log (vegetation area)	55.48	7.80	0.02	0.59	1.318	< 0.001	0.559	< 0.001	–	–
*Lepus Coreanus*	Intercept + log (vegetation area) + log (edge length)	62.70	0.00	0.87	0.75	1.476	< 0.001	1.124	< 0.001	1.595	0.016
	Intercept + log (vegetation area)	66.48	3.79	0.13	0.70	1.115	< 0.001	0.474	< 0.001	–	–
*Hydropotes inermis*	Intercept + log (vegetation area) + log (edge length)	206.69	0.00	0.90	0.98	0.170	< 0.001	0.191	< 0.001	0.331	0.010
	Intercept + log (vegetation area)	211.05	4.36	0.10	0.98	0.147	< 0.001	0.073	< 0.001	–	–
	Intercept + log (edge length)	253.92	47.23	0.00	0.94	0.106	< 0.001	–	–	0.125	< 0.001
*Sus Scrofa*	Intercept + log (vegetation area)	130.38	0.00	0.62	0.88	1.113	< 0.001	0.463	< 0.001	–	–
	Intercept + log (vegetation area) + log (edge length)	131.37	0.99	0.38	0.87	1.344	< 0.001	0.967	< 0.001	1.320	0.142

## 4. Discussion

Effective conservation of mammals requires a deeper understanding of the anthropogenic threats they face, which include expanding agriculture, urbanization, and road construction, among many others (McAlpine et al. 2006). Habitat fragmentation is a primary cause of the decreasing abundance of mammal species in urban landscapes. Moreover, low habitat quality, small habitat size, and human disturbances are key factors in the absence or low abundance of endangered species in urban forest patches (Lee et al. 2017).

In this study, both species richness and field sign frequency of mammals were associated with vegetation area. Our results demonstrate the role that the vegetation patch size and edge length play in determining the species richness and abundance of mammals in urban woods, and highlight the importance of vegetation area for the conservation of mammals in fragmented urban landscapes, given that fragmented areas cannot support the same number of species as did the original larger unfragmented habitat (Fleury and Galetti 2006). Moreover, there was lower habitat complexity in the small vegetation patches in comparison with large ones. The complexity of habitat might influence which mammal species occur there (Rocha et al. 2011).

There were different responses to vegetation area and edge length among mammal species with different habitat requirements, such as forest specialists and habitat generalists. Because leopard cats, Korean hares, and wild boars significantly prefer, and show increasing field sign frequencies in, the larger areas, these species have more affinity for large vegetation areas. Moreover, large moles and water deer might be habitat generalists, because field signs of these mammals were observed in most of our vegetation patches. They were positively correlated with vegetation area and negatively correlated with edge length in these urban woods.

Many mammals have highly specific ecological requirements, and thus may be particularly sensitive to habitat loss and fragmentation in urban landscapes. It is thus clear that conservation of mammals affected by loss and fragmentation of native forest should take into consideration both the habitat requirement of the species and the response of the species to the man-made new habitats (Rocha et al. 2011; Estavillo et al. 2013). In this study, the vegetation area was positively correlated with species richness and field sign frequency and edge length was negatively correlated. Moreover, there were species-specific relations with vegetation area and edge length. Urban land managers should take these factors into account when considering maintenance of urban vegetation patches.

Maintenance of large vegetation patches is an effective means of conserving mammals and their habitat (Galetti et al. 2009). However, the existence of a vegetation patch by itself does not guarantee higher species richness and abundance of mammals (Watling and Donnelly 2006). Habitat quality generally refers to vegetation status within a given patch, and therefore sustaining forest cover is essential for improving the quality of habitat for mammals (Laidlaw 2000; Prevedello and Vieira 2010; Lee et al. 2017). Thus, it is important to maintain not only the area of the patch, but also the type of forest cover within the patch in order to conserve the mammals and their habitats.

Since urban vegetation patches are often patchy and isolated, the spatial context of the patches can influence on the biodiversity within them (Watling and Donnelly 2006). The survival and abundance of mammals might depend on numerous habitat variables, including habitat quality, connectivity, and distance between habitat patches (Lindenmayer and Possingham 1995; Umapathy and Kumar 2000; Chetkiewicz et al. 2006; Barrantes et al. 2016). Unfortunately, spatial context of the patches such as proximity, isolation, and isolation were not analyzed in this study. Future studies should consider the spatial context of habitat patches to conserve both the habitats and the species within them in urban landscapes.
